# Native Hypovitaminosis D in CKD Patients: From Experimental Evidence to Clinical Practice

**DOI:** 10.3390/nu11081918

**Published:** 2019-08-15

**Authors:** Carlo Alfieri, Oksana Ruzhytska, Simone Vettoretti, Lara Caldiroli, Mario Cozzolino, Piergiorgio Messa

**Affiliations:** 1 Nephrology, Dialysis and Renal Transplantation, Fondazione IRCCS Ca Granda Ospedale Maggiore Policlinico, 20122 Milan, Italy; 2 Department of Clinical Sciences and Community Health, University of Milan, 20122 Milan, Italy; 3 Department of Internal Medicine n3, Ternopil State Medical University, 46002 Ternopil, Ukraine; 4 Renal Division, ASST Santi Paolo e Carlo, Department of Health Sciences, University of Milan, 20122 Milan, Italy

**Keywords:** vitamin D, mineral metabolism, CKD, cardiovascular risk, vitamin D supplementation

## Abstract

Native hypovitaminosis D (n-hVITD) is frequently found from the early stages of chronic kidney disease (CKD) and its prevalence increases with CKD progression. Even if the implications of n-hVITD in chronic kidney disease-mineral bone disorder (CKD-MBD) have been extensively characterized in the literature, there is a lot of debate nowadays about the so called “unconventional effects” of native vitamin D (25(OH)VitD) supplementation in CKD patients. In this review, highlights of the dimension of the problem of n-hVITD in CKD stages 2–5 ND patients will be presented. In addition, it will focus on the “unconventional effects” of 25(OH)VitD supplementation, the clinical impact of n-hVITD and the most significant interventional studies regarding 25(OH)VitD supplementation in CKD stages 2–5 ND.

## 1. Introduction

Vitamin D deficiency is considered to be an important public health problem. Despite the numerous warnings about the detrimental effects of native hypovitaminosis D (n-hVITD) in patients affected by chronic kidney disease (CKD), insufficient levels of 25(OH)VitD are still frequently found from the early stages of the disease [1]. Some evidence, mostly derived from experimental studies, have hypothesized a possible role for Vitamin D in the modulation of some physio-pathological processes beyond chronic kidney disease mineral bone disorder (CKD-MBD). Unfortunately, the lack of large and interventional studies focused on the so called “unconventional effects” (i.e., beyond CKDMBD) of 25(OH)VitD supplementation in CKD patients, does not conclusively demonstrate the beneficial effects of this supplementation in clinical practice. Conversely, the implications of n-hVITD in CKDMBD have been largely and well characterized in the literature, and for this reason in this review they will only be mentioned briefly [2].

Previously, our group addressed the impact of the supposed pleiotropic effects of 25(OH)VitD in renal transplanted patients [3].

Considering that the issue of the 25(OH)VitD supplementation is really important and currently debated in CKD patients, the main objectives of the present review will be:

(1). To highlight the dimension of the problem of n-hVITD in CKD stages 2–5 not dialysis (ND) patients;

(2). To present the clinical impact of n-hVITD and the most significant interventional studies regarding 25(OH)VitD supplementation in CKD stages 2–5 ND, focusing especially on the “unconventional effects” of 25(OH)VitD supplementation.

In the following sections, with the term “CKD patients” we will refer exclusively to patients affected by CKD stages 2–5 ND.

## 2. Native Hypovitaminosis D during Chronic Kidney Disease (CKD): The Size of the Problem

### 2.1. Vitamin D Metabolism and Status Categorization in CKD Patients

Vitamin D is a fat soluble pre-hormone, obtained mainly through the skin photochemical conversion of 7-dehydrocholesterol after sunlight exposition (solar ultraviolet B radiation, 290–315 nm). The 7-dehydrocholesterol undergoes a temperature-dependent rearrangement to vitamin D_3_ (cholecalciferol) [4]. Vitamin D_3_ and vitamin D_2_ (ergocalciferol) may be supplied, in minor part, also by food intake (meat, eggs, fish, liver, cod liver oil, and dairy products).

Then, vitamin D undergoes 25-α-hydroxylation in the liver, which forms 25(OH)VitD and one subsequent additional 1-α-hydroxylation primarily in renal proximal tubular cells, responsible for the generation of the active form of vitamin D, the 1,25(OH)2VitD [5].

The generation of 1,25(OH)2VitD is also regulated by the activity of the vitamin D 24-hydroxylase (CYP24A1) in renal proximal tubules, which inactivates all the active vitamin D metabolites [6].

According to 25(OH)VitD levels, native vitamin D status is normally classified as: (1) deficiency: 25(OH)VitD < 20 ng/mL; (2) insufficiency: 21 < 25(OH)VitD < 29 ng/mL; (3) sufficiency: 25(OH)VitD > 30 ng/mL [7].

Several conditions may influence 25(OH)VitD levels in humans: aging, reduced skin synthesis, reduced dietary intake, malabsorption, obesity, glucocorticoid therapy, reduced liver function, and drugs interference with vitamin D metabolism. CKD patients present generally a higher prevalence of n-hVITD compared to the general population because of additional specific alterations of the mineral and bone metabolism present in those patients from the early stages of the disease [8,9]. In addition, the association between 25(OH)VitD urinary loss and the reduction of its renal hydroxylation further implicate the native and active hypovitaminosis D typically found in these patients. A proximal renal tubular impairment in the 25(OH)VitD megalin mediated reabsorption has been experimentally demonstrated in proteinuric and megalin knock out (KO) experimental models [10,11,12]. Recent published data, by Bohnert B et al. demonstrated clearly urinary loss of both 25(OH)VitD and 1,25(OH)2VitD and vitamin D binding protein in experimental nephrotic syndrome [13]. In addition, the low renal 1-α-hydroxylation capability, determined by the reduction of nephron mass, and the effects of phosphorus, FGF23 and αKlotho on the cytochromes CYP27B1 and CYP24A1 determine the additional decrease of 1,25(OH)2VitD [14].

### 2.2. Native Hypovitaminosis D Epidemiology in CKD Patients

The prevalence of n-hVitD in CKD has been evaluated in a monocentric American study, performed on a total of 43 CKD patients in which 86% of patients did not have sufficient 25(OH)VitD levels. Of note, 42% of them had 25(OH)VitD insufficiency, and 42% had mild 25(OH)VitD deficiency. Severe vitamin D deficiency (<5 ng/mL) was found in the remaining 2% of patients [1]. Recent data obtained in an Italian experience, including 450 prevalent CKD patients, report a mean level of 25(OH)VitD of 17.5 ± 11.2 ng/mL, and the presence of deficiency or insufficiency respectively in 66.4% and 16.5% of patients. In this cohort, the presence of diabetes mellitus was related to lower 25(OH)VitD levels. An inverse correlation between 25(OH)VitD and age, Parathormone (PTH), urinary protein excretion and Charlson index was also found, whereas a direct correlation with hemoglobin was demonstrated. In multivariate analysis only age and PTH levels impacted independently on 25(OH)VitD levels [15].

In our department, in Milan, we analyzed retrospectively 133 incident CKD stage 3–4 outpatients (males = 95; age 77 ± 11 years; eGFR-EPI 24 ± 10 mL/min). At the first evaluation, the mean levels of 25(OH)VitD were 17.5 ± 11.2 ng/mL. Not sufficient 25(OH)VitD levels, despite the native vitamin D supplementation prescribed in the 72% of patients, was found in 61% of patients, 42% had vitamin D insufficiency, and 19% had vitamin D deficiency. Interestingly, in our cohort, we did not find any difference in 25(OH)VitD according to the supplementary therapy. This result might support the evidence of the frequent inability to reach sufficient 25(OH)VitD levels in CKD patients with the simple supplementation. In this sense, a correction of life and alimentary habits can be important [16].

### 2.3. Native Vitamin D Supplementation: Dose, Interval,Toxicity

Despite the most important international guidelines suggesting 25(OH)VitD supplementation in presence of n-hVITD, at the moment the optimal strategy of supplementation and the optimal levels of 25(OH)VitD to reach are still debated worldwide. What KDIGO guidelines suggest, is that deficiency and insufficiency of 25(OH)VitD in CKD patients should be treated through strategies provided to the general population. No special considerations are present concerning which form of native vitamin D to use, and there is no strong evidence about a superiority in particular habits of dose and interval schedules [17]. In 2016, the Italian Society of Nephrology published, a report suggesting a supplementation in CKD patients with 25(OH)VitD < 30 ng/mL, using the scheme that nephrologists are most confident with. At the moment three different forms of native vitamin D are available: ergocalciferol, cholecalciferol and calcifediol. Among them, cholecalciferol seems to be superior in increasing 25(OH)VitD levels, with a safe dose of 10,000 UI/week [18,19]. A novel extended-release (ER) calcifediol formulation has been recently tested in 429 CKD patients. After a randomization 2 (daily pills containing oral ER calcifediol, 30 or 60 mcg):1 (placebo once daily), the cohort was followed for 26 weeks. In the sequent open label extension study, ER calcifediol was prescribed for an other 26 weeks in 298 patients. This study demonstrated a good correction of n-hVITD, a good effect on PTH, and a safe profile for oral ER calcifediol [20]. This formulation might in the future solve the problems of the adherence to the 25(OH)VitD therapy, burdened by the drop formulation and by the unusual assumption schemes prescribed [21]. Independently of the native form of vitamin D used, clinicians should pay close attention to vitamin D toxicity, avoiding too high levels of plasmatic 25(OH)VitD, and monitoring calcium and phosphorus levels. There should be particular attention during summer season, when the sunlight exposition is significantly higher [22]. According to our experience, levels of 25(OH)VitD of 30–60 ng/mL might represent a good general target in CKD population. Higher levels might be suggested to obtain effects on immunity, proteinuria etc., but unfortunately the little evidence and also the high risk of toxicity limit this suggestion. We agree with the Italian Nephrology of Society indications about the discontinuation of native vitamin D supplementation in presence of 25(OH)D levels >100 ng/mL and/or presence of hypercalcemia (Ca > 10.4 mg/dL) in the absence of active vitamin D therapy.

## 3. Native Hypovitaminosis D in CKD: Experimental and Clinical Evidence

**Figure 1:**
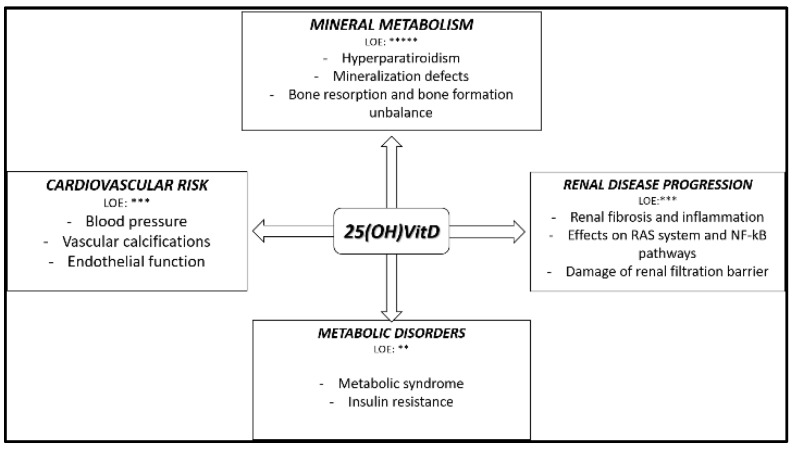
Principal effects and their level of evidence of native hypovitaminosis D in chronic kidney disease (CKD) patients. LOE: level of evidence. ** slight level of evidence; *** moderate level of evidence; ***** strong level of evidence;

The principal circulating form of vitamin D is 25(OH)VitD. In the early 2000s, the presence of 1-α-hydroxylase enzyme was noted in many tissues outside the kidneys, and a certain binding affinity of 25(OH)VitD for vitamin D receptor (VDR) was demonstrated [23,24]. Almost all tissues and cells, including brain, heart, skeletal muscle, smooth muscle cells, pancreas, activated T and B lymphocytes, and monocytes express the vitamin D receptor (VDR) [25]. Consequently, much experimental evidence has implicated the hypovitaminosis D (25(OH)VitD and/or 1,25(OH)2VitD) and the reduced activation of VDR in the modulation pathological processes also beyond CKDMBD [26]. The principal effects and their level of evidence (LOE) of n-hVitD in CKD patients are summarized in Figure 1.

In the following paragraphs, the indications of the impact of vitamin D status in CKD in mineral and bone disorders, cardiovascular risk, metabolic disorders, renal disease progression and patients outcome, mostly based on experimental, retrospective and association studies, will be presented.

### 3.1. Native Hypovitaminosis D and Mineral Metabolism

The principal, and most recognized function of vitamin D has been historically related to mineral metabolism and bone health. Vitamin D acts by a modulation of the intestinal and renal absorption of calcium and phosphate, and inhibiting PTH production. During CKD, changes in calcium and vitamin D metabolism may promote bone mineralization and have an important role in the pathogenesis of osteoporosis in patients with CKD [27,28]. The relationship between n-hVitD and CKDMBD alterations, independently of the active form effects of the vitamin, has been well investigated in CKD stages 2–5 ND patients. In 2014, a study performed on 6949 CKD patients evaluated the combined effects of 25(OH)VitD deficiency and CKD on bone mineral density. A significant increased risk of osteoporosis or osteopenia, both at femur neck and total hip level, was present and associated with elevated levels of PTH and with n-hVitD. More recently, the favorable effect on many biochemical parameters of CKD-MBD (as secondary hyperparathyroidism, changes in bone turnover markers, and a favorable trend in FGF-23 levels) of the n-hVitD correction in pre-dialysis CKD patients was evidenced, indicating n-hVitD as a potential independent risk factor for the development of CKD-MBD [29].

### 3.2. Native Hypovitaminosis D and Metabolic Disorders

Vitamin D deficiency had been associated with metabolic disorders, as metabolic syndrome and insulin resistance (IR). The association between 25(OH)VitD and metabolic syndrome in CKD patients was explored in 495 patients. The prevalence of metabolic syndrome increased as 25(OH)VitD levels declined. In addition, in multivariate analysis after a correction for several confounding factors, 25(OH)VitD resulted in being the most independent factor influencing the risk of metabolic syndrome [30]. The association of kidney function with 25(OH)VitD and some components of the metabolic syndrome had been tested in 2007, in 14,679 participants of the Third National Health and Nutrition Examination Survey (NHANES III). Insulin resistance (IR) was estimated by means of Homeostasis Model Assessment (HOMA) -index calculation. After a stratification of the participants according to CKD stage and quartile of 25(OH)VitD and correction for potential confounding factors, CKD stage and quartile of 25(OH)VitD were inversely associated with HOMA-index and fasting insulin, indicating a potential independent association between those two factors and IR [31] The lower activation of VDR in the pancreas and in skeletal muscle in the presence of n-hVitD might, in part, explain these observations [32]. A confirmation of the relation between n-hVitD and IR was recently obtained in 100 type 2 diabetes patients with CKD stages 3–4, in which glycated hemoglobin was negatively correlated with 25(OH)VitD. Of note, 25(OH)VitD was also negatively correlated with total daily dose of insulin prescribed [33].

### 3.3. Native Hypovitaminosis D and Cardiovascular Risk

Much interest has been expressed recently concerning the influence of 25(OH)VitD in cardiovascular risk. Of note, although experimental data are almost convincing about the beneficial effect of 25(OH)VitD in cardiovascular structures, data from human studies, especially prospective, are still contradicting each other. In experimental models, the presence both of the vitamin D receptor (VDR) and of the 1-α-hydroxylase has been reported in cardiovascular tissues [34]. The most important evidence available was based on the effect of the activation of VDR by the active forms of vitamin D, whereas data concerning the direct effects of 25(OH)VitD are still scanty. The importance of VDR activity in the endothelium was proven by the strong vascular calcifications present in VDR knockout (KO) mice aorta [35]. In hypertensive models, endothelial VDR activation seems also to act as a modulator of vascular endothelial growth factor (VEGF) activity, affecting calcium influx across the cell membrane, as well as endothelium-dependent vascular smooth muscle contractions and vascular tone [36]. In a more recent study by Painzo et al. the beneficial effect on myocardial structure of VDR activation was verified [37]. Vitamin D effects on the growth, differentiation and proliferation of cardiomyocytes were also experimentally tested [38] Despite this experimental evidence, it is at the moment difficult to understand if the potential benefit of VDR activation might be 25(OH)VitD directly mediated or rather, the presence of extrarenal 1-α-hydroxylase may mediate a local production of active vitamin D as a direct and exclusive promotor of cellular local effects [39]. Clinical associative studies performed in CKD patients are still poor and sometimes contrasting. In 2007, Forman et al. reported in the general population a significant inverse correlation between 25(OH)VitD and the risk of incidents of hypertension [40]. In a cohort of pediatric CKD patients, an increase in cardiovascular disease risk, which tends to manifest in childhood, was found in the presence of hypovitaminosis D [41]. An independent and negative association between 25(OH)VitD and vascular calcification, evaluated by the Kauppila index (KI), was found in 210 CKD stages 4 and 5 ND patients in a Spanish study published in 2010. In that study, in multivariate analysis, 25(OH)VitD resulted an independent predictor of KI > 3 (*p* = 0.02) and KI > 7 (*p* = 0.017) [42]. The association between n-hVit D and endothelial dysfunction was explored in a study published in 2016 by Luo et al. in which 283 CKD patients were studied to evaluate the association between 25(OH)VitD and arterial stiffness, an early marker of systemic atherosclerosis. In multivariate analysis, 25(OH)VitD resulted independently related with brachial-ankle pulse wave velocity [43]. In another study, 117 CKD patients underwent ultrasound to test endothelial function by brachial artery flow-mediated dilation (FMD). In the same cohort, 25(OH)VitD, soluble vascular cell adhesion molecule-1 (sVCAM-1) and sE-selectin were dosed. After the adjustment for confounding factors, FMD was significantly lower in patients with low 25(OH)VitD. Compared to patients with sufficient levels of 25(OH)VitD, in n-hVitD patients, sVCAM-1 and sE-selectin were higher [44]. Recently, a lot of interest has been attributed to the possible role of calciprotein particles (CPPs) and matrix vesicles (MVs) in vascular calcification modulation. According to recent data, in fact, vitamin D might directly stimulate the vitamin K-dependent matrix-Gla-protein production, resulting in a reduction of vascular calcification development and progression [45] 

Some recent experimental data have also hypothesized a potential interaction between Vitamin D and calcimimetics by means of the reciprocal interaction with their receptors in modulating vascular calcifications processes and consequently cardiovascular risk [46]. In the study published by Mary et al. for example, an increase in the calcium-sensing receptor (CaSR) expression was described after the exposition of human vascular smooth muscle cells (VSMC) to calcitriol concentrations within 0.5 and 5 nmol/L. Interestingly, in the presence of pro-calcification conditions, the treatment of the cells with a low dose of active vitamin D (1.0 nmol/L) resulted in a reduction of the Ca-induced decrease of CaSR expression and a protection against human VSMC mineralization [47]. Despite several comparison studies between Vitamin D and calcimimetics, nowadays the clinical evidences of the beneficial effect on the major clinical outcomes during CKD of the contemporary administration of the two drugs is still limited. Future studies about this topic in this specific subgroup of patients are desirable.

An association between n-hVitD and subclinical cardiac damage was reported by an Italian group. Sixty-seven patients with CKD (eGFR  ≥  30  mL/min) and 15 healthy controls matched for age and sex were examined. The principal aim of the study was to examine the association between insulin resistance (IR), 25(OH)VitD and left ventricular hypertrophy (LVH). In univariate analyses, both IR and n-hVitD resulted in being independently related to LVH and atherosclerotic disease. It is important however to specify that in both groups considered, 25(OH)VitD levels were >30 ng/mL, with CKD patients that have significant lower levels of the vitamin (30.7 ± 19.0 ng/mL vs. 41.7 ± 14.6 ng/mL, *p* = 0.03) [48]. This might suggest the need of a better definition of 25(OH)VitD target levels in CKD patients.

### 3.4. Native Hypovitaminosis D in Animal Model with Kidney Disease

The renal protective effect of Vitamin D has been hypothesized after some experimental evidence and after the finding of the VDR presence in all the cell types of the kidney. In a model of unilateral ureteral obstruction (UUO), for example, active vitamin D treatment reduced renal fibrosis and inflammation [49]. In the paper published by Ana de Bragança et al. the general and renal status and the histological lesions in 5/6 nephrectomized rats which underwent two different 25(OH)VitD dietary regiments were investigated. Compared to 5/6 nephrectomized rats receiving standard diet (VD+), those 5/6 nephrectomized rats fed with 25(OH)VitD-free diet (VD-) were affected by higher blood pressure, and had a stronger decline in renal function and a significantly worse CKDMBD control. Moreover, VD- had a higher degree of tubulointerstitial fibrosis, a higher expression of inflammation and fibrosis remodeling [50].

Experimental studies have demonstrated that VDR activation may interfere with the modulation of different pathologic pathways such as the renin-angiotensin system (RAS) and the NF-kB. All of them, acting in combination, are crucial in the development and maintenance of renal inflammation and fibrosis, independently of the basilar nephropathy. The increased activity of RAS present during CKD has a broad range of pathogenic consequences and promotes renal injury [51] The relationship between vitamin D and RAS was studied by means of KO-VDR mice, in which a severe proteinuria, glomerulosclerosis and interstitial fibrosis was found. Unfortunately, most of the experimental and clinical research explored the effect of the use of active vitamin D or vitamin D analogs in reducing RAS activity and renal effects in different models of CKD and not the direct effect of 25(OH)VitD. In a UUO-VDR-null mice (VDR-) model, in 2010, Zhang et al. explored the effect of VDR in renal fibrogenesis. Compared to wild type mice (WT), VDR- had a higher degree of renal damage (i.e., marked tubular atrophy and interstitial fibrosis). In addition, in VDR+ a significant induction of extracellular matrix proteins (fibronectin and collagen I), profibrogenic and proinflammatory factors (TGF-β, connective tissue growth factor, and monocyte chemoattractant protein 1), and an epithelial-to-mesenchymal transition accompanying this histologic damage was found. Immunostaining showed marked accumulation of AngI/II in the interstitium of the VDR- kidney. Those differences resulted partially reversible after the reduction of RAS activity obtained with losartan therapy, suggesting a direct role for angiotensin II in enhanced renal fibrosis in VDR-kidneys [52]. In addition to the potential beneficial effect of VDR activation on inflammation and fibrosis, some experimental findings have reported also that VDR activation may interfere with the damage of the glomerular filtration barrier present in some proteinuric nephropathies. Diabetes has been the experimental model most used to investigate the VDR effects on podocytes. In a transgenic diabetic experimental model, performed in 2012 by Wang et al. it was evidenced that vitamin D-VDR signaling in podocytes plays a critical role in the protection of the kidney. In particular, the authors evidenced that active vitamin D, by its interaction with VDR, was able to suppress high-glucose–induced apoptosis of podocytes by blocking p38- and ERK-mediated pro apoptotic pathways. More recently, the effect of 1,25-dihydroxyvitamin D3 (calcitriol) and paricalcitol on podocytes morphology and survival was investigated in a diabetic animal model. The results obtained both on cellular and in vivo experiments demonstrated better podocytes survival restoration of nephrin signaling in the presence of VDR activation [53].

### 3.5. Native Hypovitaminosis D and Immune System in CKD

The relationship between 25(OH)VitD and the immune system has recently been the subject of much attention deriving from the evidence of finding VDR in the immune system cells, especially macrophages, monocytes, T-cells, B-cells and dendritic cells [54]. As for the other topics, is difficult to conclude if in immune system cells 25(OH)VitD has a direct or a 1,25(OH)2VitD mediated effect. In vitro studies demonstrated the capability of macrophages, monocytes and dendritic cells, to convert by means of their 1α-hydroxylase activity 25(OH)VitD in the active form [55,56]. In agreement, in 2009, Adams et al. showed clearly that in vitro 25(OH)VitD treatment of monocytes from 25(OH)VitD deficient patients increases the 1,25(OH)2D synthesis [57]. Moreover, recent evidence suggests a vitamin D modulatory effect on macrophage adhesion and infiltration in presence of pathologic processes [58,59].

What has been deeply investigated, is the effect of vitamin D on the transcription factor family NF-kB, that has a primary role in the regulation of immune response in renal pathologic processes [60]. In 2005, Xu et al. investigated the effects of cholecalciferol pretreatment on renal inflammation during lipopolysaccharide (LPS)-induced nephropathy, a model of experimental sepsis-induced acute kidney injury. Cholecalciferol was administered in the group at the dose of 25 μg/kg, at 1, 24, 48 h before LPS intraperitoneal injection. Interestingly, the cholecalciferol treated group had a lower reduction of renal function and less histological damage. Renal inflammatory cytokines, chemokines and adhesion molecules also were reduced in the cholecalciferol-treated group. The experiments performed by the authors, demonstrated a direct activation of the VDR by cholecalciferol and a consequent repression of the LPS-induced nuclear translocation of NF-κB p65 subunit in the renal tubules [61]. Analogous results in the same experimental model have been obtained more recently using a pretreatment with Paracalcitol [62]. The beneficial VDR activation effects on inflammation have been also demonstrated in chronic models of renal diseases such as UUO, diabetes and toxic [63].

Unfortunately, at the moment, studies about the influence of 25(OH)VitD in immune system of CKD 2-5ND patients are still missing. In any case, some data are present in dialysis population. In 2016, Meireles et al. observed a decrease in circulating inflammatory markers and an increase in monocytes VDR expression of 38 hemodialysis nHVitD patients after the specific. 25(OH)VitD supplementation [64].

### 3.6. Native Hypovitaminosis D and Mortality in the Presence of CKD

Data reported by experimental studies found some confirmation also in clinical studies. For example, in 2009, Zehnder et al. demonstrated in 174 patients with a variety of kidney diseases an association between renal inflammation and decreased serum vitamin D metabolites, both 1,25(OH)2D or 25(OH)VitD, involving the activation of the paracrine/autocrine vitamin D system [65].

More debated is the relationship between 25(OH)VitD and mortality in CKD patients. In 2011, a metanalysis performed by Pitz et al. explored the 25(OH)VitD serum concentrations in relation to all causes of mortality of CKD patients. Effectively, considering the 10 trial examined in this metanalysis, a significant decrease in mortality with increasing serum 25(OH)VitD levels was observed. In particular, an increase of 10 ng/mL reduced the mortality risk by 14% [66]. Of note, in only three studies considered were there present only patients with CKD stages 2–5 ND. Among them, the biggest was the study published by Mehrotra et al. in 2009, in which 3011 patients studied between 1988–1994 were recruited. A relationship between low serum 25(OH)VitD and the risk of death among CKD patients was found [67]. Levels of 25(OH)VitD and supplementation with oral active Vit D3 cholecalciferol were also evaluated in association with morbidity and mortality in 516 patients with CKD in 2016 in the paper published by Namir et al. In their study the authors showed a prevalence of severe vitamin D deficiency (<5 ng/mL) of 34%, whereas in almost 50% of the cohort studied vitamin D insufficiency was present. Native vitamin D levels below 15 ng/mL were correlated with renal outcomes (hazard ratio (HR) 3.17, 95% CI 1.12–8.94). The supplementation with cholecalciferol ameliorated the combined renal outcomes and death in univariate analyses, with a 25% reduction in mortality (HR 0.755 (95% CI 0.54–1.00) (*p* = 0.02) each 10 ng/mL of 25(OH)VitD increase [68]. It is important to specify, however, that in none of the works mentioned, a correction for PTH and CKDMBD parameters modification was made.

According to the studies presented in the previous paragraphs we can conclude that despite the well characterized effects of 25(OH)VitD on CKDMBD, which at the moment in our opinion represent the only clear clinical indication of 25(OH)VitD replacement, some discrepancy between experimental and clinical studies is present concerning the unconventional effects of 25(OH)VitD, especially in cardiovascular protection and mortality in CKD patients.

For this reason, the development of randomized interventional studies will be important in the future. In the next paragraphs the most impacting randomized interventional studies available at the moment will be presented.

## 4. Native Vitamin D Supplementation in CKD Patients: Evidence from Randomized Interventional Studies

Despite the numerous data of the association previously reported, data derived by randomized prospective trials on the effect of native vitamin D supplementation on major clinical outcomes are still scanty and discordant with each other. The principal characteristics and results of the recent randomized interventional studies in patients with CKD stages 2–5 ND are summarized in Table 1. In 2002, by means of a randomized, double-blinded, placebo-controlled study, the ability of native vitamin D supplementation in delaying the onset of secondary hyperparathyroidism was tested in 72 children with CKD stages 2–4. No baseline differences were present between the two groups in 25(OH)VitD levels with a relatively native vitamin D insufficiency present in both groups (treated group 20.1 ± 7.1 ng/dL vs. placebo group 20.8 ± 7.2 ng/dL *p* = 0.69). Patients were followed up for 24 months. During the follow up time, in 9 of 20 children on placebo and 3 of 20 children on ergocalciferol treatment the development of hyperparathyroidism was found (odds ratio = 4.64, 95% confidence interval = 1.02−21.00), with a significantly longer time to the development of the anomaly in treated children (hazard ratio = 0.30, 95% confidence interval = 0.09–0.93, *p* = 0.05) [69].

In 2012, an 8-week randomized, placebo-controlled, double-blind parallel intervention study was conducted in hemodialysis (HD) and no-HD CKD patients. The treated group received 40,000 IU of cholecalciferol orally per week. In no-HD CKD patients (*n* = 26), by contrast with HD patients, the increase of 25(OH)VitD corresponded to a significant rise of 1,25-OHD (*n* = 13, *p* < 0.01) and a lowering of PTH (*n* = 13, *p* < 0.001). However, despite the modifications of CKDMBD parameters, the supplementation of native vitamin D had no effects on endothelial and inflammatory markers, blood pressure and arterial stiffness parameters [70]. The clinical effect of native vitamin D supplementation has been explored also more recently in two randomized studies. In their work, Levin et al. studied 119 patients with an eGFR of 15–45 mL/min per 1.73 m^2^ and evaluated the modification in pulse wave velocity after 6 months of treatment with a fixed dose of oral calcifediol (5000 IU), calcitriol (0.5 µg), or placebo. By contrast with the previous study, in this case a reduction of pulse wave velocity (PWV) was found only in the calcifediol group whereas a stability and a rise was found in the calcitriol and the placebo groups respectively. In addition, patients in the highest 25-hydroxyvitamin D tertile had significant decreases in PWV compared to the other tertiles at the end of follow up [71]. In the same year, Kumar et al. explored the effect of cholecalciferol supplementation (300,000 IU) on vascular function in 120 patients with CKD stage 3–4 and vitamin D deficiency. Patients were randomized by a 1:1 ratio to receive, at baseline and after 8 weeks, either two directly observed oral doses of cholecalciferol or placebo. At 16 weeks of observation, compared to the placebo group, the treated patients showed a significant increase in endothelium-dependent brachial artery flow-mediated dilation, associated to a reduction of/in? arterial stiffness and of circulating IL-6 levels. No analysis related to cardiovascular risk was assessed [72]. Recently, a randomized, placebo-controlled trial, performed in the general population (25,871 participants followed up for 5.3 years) has been published. Treatment consisted in 2000 IU of cholecalciferol per day and did not affect cardiovascular endpoints [73]. Finally, the results of the J-DAVID study were published at the beginning of 2019. In this randomized open-labels study performed in Japan, 976 patients receiving maintenance hemodialysis with serum intact parathyroid hormone levels less than or equal to 180 pg/mL were randomized to one of the two treatment arms: treatment with oral alfacalcidol or treatment without using any VDRA and followed up for 48 months. Composite of fatal and non-fatal cardiovascular events were chosen as primary outcome. Also in this case, as with the PRIMO and OPERA studies active vitamin D did not reduce the risk of a composite measure of select cardiovascular outcomes [74]. No dedicated notable randomized controlled trials have been realized to explore the effects of vitamin D supplementation on survival, cardiovascular disease (CVD) and hospitalization rates in CKD patients 2–5 ND. The only data available on this issue derive from metanalyses, frequently burdened by poor statistical power [75,76]. Finally, despite experimental results, in prospective trials involving CKD patients from different nephropathies, native vitamin D supplementation gave contrasting results in determining a significant reduction of urinary protein excretion [77,78].

## 5. Conclusions and Future Perspectives

In our review we point out the attention given to the principal evidence of beneficial effects of 25(OH)VitD in CKD stages 2–5 ND patients. The principal conclusions of our review are:

- Concerning the control of CKDMBD parameters, and only on this issue, both experimental, association and interventional data agree on the fact that 25(OH)VitD has a crucial role. Consequently, in our opinion, 25(OH)VitD should be used as primary choice, when indicated, in the treatment of CKDMBD, and, in cases of severe hypovitaminosis, a supplementation, when possible and safe, might be suggested independently to the other CMKMBD parameters.
- The results from interventional studies are discordant concerning beneficial effects, beyond CKDMBD, of 25(OH)VitD supplementation. It is important to underscore, however, that at the moment we are not able to define the optimal level of 25(OH)VitD that should be maintained in CKD patients, so we still refer to recommendations for the general population. Hopefully, future more focused and more interventional studies might add some knowledge indicating and “personalizing” the target 25(OH)VitD levels.
- Native Vitamin D supplementation does not exclude the use of the active form of vitamin D. So, in agreement with what the guidelines support, the two forms should be considered not in contrast but potentially together in the treatment of secondary hyperparathyroidism and, probably, in bone health. More trials in the future might provide information about the lack of classical effectiveness of 25(OH)VitD supplementation in CKD patients in clinical practice.

## Figures and Tables

**Table 1:** Principal characteristics and results of the recent randomized interventional studies in patients with chronic kidney disease (CKD) at stages 2–5 not dialysis (ND).

Type of Patients	Number of Patients	Type of Intervention	Results	Reference
CKD stages 2–4	87	GrA: Cholecalciferol 5000 IU/wk; GrB: Cholecalciferol 20,000 IU/wk	In GrB vs. GrA: significant increase of 25OHVitD, 1,25OHD2 and reduction PTH	Oksa et al., 2008
CKD stages 1–5	25	GrA: Cholecalciferol 40,000 IU/wk; GrB: placebo	In GrA vs. GrB: significant increase of 25OHVitD, 1,25(OH)D2, FGF23 and reduction PTH	Markmann et al., 2012
CKD 3–4	38	GrA: Ergocalciferol 50,000 IU/wk for one month followed by 50,000 IU/mth for 5 mths; GrB: Placebo	In GrA vs. GrB: significant increase of 25(OH)VitD, endothelium dependent microcirculatory vasodilatation and reduction of pulse pressure. No effects on PWV and LVMI	Dreyer et al., 2014
CKD stages 3–4	429	Study 1 (n = 213): GrA: ER Calcifediol 12 wk at 30 g/daily + 14 wks 30 or 60 g/daily; GrB 26 wks placebo Study 2 (n = 216): GrA: ER Calcifediol 12 wks 30 g/daily + 14 wks 30 or 60 g/daily; GrB: 26 wks placebo	In GrA vs. GrB in both studies: significant increase of 25OHVitD and reduction PTH	Sprague et al., 2016
CKD 3–5	44	GrA:Cholecalciferol 50.000 IU/wk; GrB: Ergocalciferol 50.000 IU/wk	In GrA vs. GrB: significantly higher increase of 25(OH)VitD. No differences in 1,25(OH)D2 and PTH	Weltmore et al., 2016
CKD stages 3–4	128	GrA: Cholecalciferol 2000 IU/day; GrB: Calcitriol 0.5 g/day	In GrA vs. GrB: significant increase of 25OHVitD in GrA vs. GrB. No effects on FMD	Kendrick et al., 2017
CKD stages 3–4	120	GrA: Cholecalciferol 300,000 UI mth; GrB: Placebo	In GrA vs. GrB: significant increase of 25OHVitD, 1,25OHD2 and reduction PTH. Increase of FMD and PWV	Kumar et al., 2017
CKD stages 3–4	119	GrA: Placebo; GrB: Calcitriol 0.5 g 3×/wk; GrC: Calcifediol 5.000 IU 3×/wk	In GrC vs. others: significant decrease of PWV. Patients in the highest 25(OH)VitD tertile at trial end had significant decreases in PWV	Levin et al., 2017

Footnotes: GrA: group A; GrB: Group B; GrC: Group C; wk: week; wks: weeks; mth: month; mths: months; PWV: pulse wave velocity; FMD: flow mediated dilation; LVMI: left ventricular mass index. PTH: Parathormone.
